# Fabrication of Porous Ag/TiO_2_/Au Coatings with Excellent Multipactor Suppression

**DOI:** 10.1038/srep43749

**Published:** 2017-03-10

**Authors:** Duoduo Wu, Jianzhong Ma, Yan Bao, Wanzhao Cui, Tiancun Hu, Jing Yang, Yuanrui Bai

**Affiliations:** 1 College of Chemistry and Chemical Engineering, Shaanxi University of Science & Technology, Xi’an 710021, China; 2 College of Bioresources Chemical and Materials Engineering, Shaanxi University of Science & Technology, Xi’an 710021, China; 3 Science and Technology on Space Microwave Laboratory, China Academy of Space Technology, Xi’an 710100, China

## Abstract

Porous Ag/TiO_2_/Au coatings with excellent multipactor suppression were prepared by fabrication of porous Ag surface through two-step wet chemical etching, synthesis of TiO_2_ coatings by electroless-plating-like solution deposition and deposition of Au coatings via electroless plating. Porous structure of Ag surface, TiO_2_ coatings on porous Ag surface and Au coatings on porous Ag/TiO_2_ surface were verified by field-emission scanning electron microscopy, the composition and crystal type of TiO_2_ coatings was characterized by X-ray photoelectron spectroscopy and X-ray diffraction. Secondary electron yield (SEY) measurement was used to monitor the SEY coefficient of the porous Ag coatings and Ag/TiO_2_/Au coatings. The as-obtained porous Ag coatings were proved exhibiting low SEY below 1.2, and the process was highly reproducible. In addition, the porous Ag/TiO_2_/Au coatings showed excellent multipactor suppression with the SEY 1.23 and good environmental stability. It is worth mentioning that the whole preparation process is simple and feasible, which would provide a promising application in RF devices.

Multipactor is a potentially detrimental phenomenon in RF devices in space, such as high-energy particle accelerators^1^ gyroklystron^2^ waveguide filters^3^ and so on. Multipactor would cause many serious problems, like disordering the resonance equipment, generating narrow band noise near the carrier frequency, causing the component performance to decrease and even making the system invalid. Multipactor discharge is caused by electron multiplication due to emission of secondary electrons from materials surface, resulting from the impact of the accelerated electrons with energy in RF field. Therefore, several approaches are carried out to prevent multipacting by reducing the secondary electron yield (SEY). Researchers have focused on thin coatings with a reliable low SEY such as carbon nitride^4^ amorphous carbon layers^5^ and titanium nitride^6^. The beneficial effect is usually attributed to the lower SEY of sp^2^ carbon with respect to other hybridization states. Although the carbon and carbon nitrides are known to have a low SEY, they are generally believed to be applied in accelerators for the poor electrical conductivity.

On the other hand, porous materials that can effectively trap escaping secondary electrons, are prepared to suppress multipactor. Porous materials include porous metals^7^, porous metal-oxide semiconductors^8^, porous carbons^9^,^10^,^11^, etc. And according to physical state of porous materials, it can be classified into three different categories, namely, powder, block and film. Porous Ag film has been demonstrated as promising candidates for multipactor suppression in RF devices among various porous materials. Ag is most frequently used as coating material in RF devices, which results from its best electro-conductibility and lower SEY coefficient. Porous architecture films have fascinating physicochemical properties and provide ample opportunities for secondary electrons to enter and undergo multiple reflections, therefore secondary electrons can be suppressed. Meanwhile, compared to bulk silver materials, film is low cost. Up to now, common methods such as template method^12^ and solution-phase approach^13^ are mainly direct to prepare porous powder. However, porous Ag film with micron scale pores has been reported in few articles. M. Ye *et al*. have fabricated porous Ag surface to cause strong SEY suppression effect via Fe(NO_3_)_3_ solution etching^14^. Preparing the silver trap structure by using 1-D ZnO nanoarrays as building blocks on the plated silver exhibited a 30.8% reduction of SEY in our earlier study^15^. Several simulation and experimental results have demonstrated that high aspect ratio and large porosity of porous Ag film are capable of avoiding the second electron emission. However, since silver is susceptible to oxidation, the environmental stability of porous Ag surfaces is relatively poor.

In order to keep from oxidation and improve environmental stability, forming a protective layer on Ag surface is necessary. Gold coatings with low surface resistance, low SEY, and stable chemical and physical structure, on a rough silver surface are considered as a viable alternative^16^, whereas that will probably be formed into gold-silver alloy, which is unbeneficial to RF devices. Accordingly, transition layer is essential. Titanium dioxide(TiO_2_) film was chosen to be synthesized as transition layer prior to the deposition of Au coatings on porous Ag surface for several reasons, including its low cost, electronic properties, and easiness of thin film preparation.

Nowadays, many works have been performed on preparation of TiO_2_ films, including physical and chemical methods, such as magnetron sputtering method^17^, atomic layer deposition^18^, sol-gel method^19^, electroless-plating-like solution deposition (EPLSD) approach^20^,^21^, *etc*. Wu L. Z. *et al*. reported a simple and mild chemical method for synthesis of metal/TiO_2_ (M = Au, Ag) transparent aqueous sols and a corresponding EPLSD procedure for the preparing flexible metal/TiO_2_ antibacterial film on the polymer substrates. In the EPLSD process, a metal/TiO_2_ nanocomposite sol should be achieved, the as-synthesized composite was covered with the un-decomposed peroxo group. Secondly, the aniline absorbed PET film was dipped into the metal/TiO_2_ nanocomposite solution, a radical polymerization was triggered by the peroxo group, and the produced polymer would play a role in attaching metal/TiO_2_ nanocomposite to the PET surface as a self-binder reagent. Though the EPLSD procedure is simple and cost-effective, it is limited to polymer substrates, and it is difficult in realizing conformal deposition and obtaining dense film. In the present report, we developed an available method to fabricate TiO_2_ films on porous Ag surface, which is also called the EPLSD, but the preparation mechanism is different from that of the above approach. The EPLSD we reported would achieve conformal and dense TiO_2_ thin films.

As for preparation of gold films, many approaches have been successfully put into practice, such as magnetron sputtering^22^ electrodeposition^23^ electroless plating (ELP)^24^ *etc*. ELP is a feasible approach, which is based on the deposition and reduction of metallic ions from a solution to a surface without applying an electrical potential. ELP has many advantages. Firstly, it doesn’t require any expensive or complicated equipment. Secondly, it can provide uniform surface coverage on multidimensional surfaces without the directional limitation encountered during evaporation or sputtering. A method to directly electroless plating thin gold films on planar, curved line-of-sight-obscured silicon nitride surfaces was developed^25^. The sequence of the method is compelling: directly sensitizing the silicon nitride substrate with a Sn^2+^ solution, activation by deposition of an elemental silver layer by oxidizing the surface Sn^2+^ to Sn^4+^ and reducing Ag^+^ to elemental silver, and finally gold plating by galvanic displacement of the silver with reduction of Au^+^ to Au^0^. Sensitization and activation are indispensable to eletroless plating for polymers and silicon nitride, but no established prior art for electroless plating of gold onto TiO_2_. Taking advantage of catalytic activity of TiO_2_, we presented a dramatically simplified electroless gold deposition method onto TiO_2_ surface in the study, the gold coatings were conformally deposited on the TiO_2_ surface.

Figure 1 summarizes the overall synthetic procedure adopt to obtain porous Ag/TiO_2_/Au coatings in this study. The procedure was mainly related to three phases: two-step wet chemical etching of silver plated aluminum alloy to achieve appropriate porous Ag coatings with low SEY coefficient, then electroless-plating-like solution deposition of TiO_2_ coatings to obtain a transition layer, and electroless plating Au on porous Ag/TiO_2_ surface to get a stable overlayer for multipactor suppression.

## Results

### Performance of porous Ag surface synthesized by wet chemical etching

**Figure 2:**
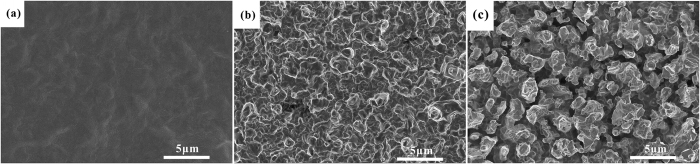
SEM images of the original plated Ag sample (**a**), the Ag sample etched by acid mixture (**b**) and the porous Ag surface etched by acid mixture and ferric nitrate aqueous solution sequentially (**c**).

SEM images of the samples before and after wet chemical etching are displayed in Fig. 2. The surface morphology of the original plated Ag sample (see Fig. 2(a)) is relatively smooth, without obvious silver grains. The sample etched by acid mixture (see Fig. 2(b)) presents dense silver grains in the range of microns which provides effective grain boundary. Figure 2(c) shows the porous Ag surface morphology of the sample etched by acid mixture and ferric nitrate aqueous solution sequentially. It is discovered that the micro-pores on the Ag surface (see Fig. 2(c)) are formed along the grain boundary of the sample etched once (see Fig. 2(b)). This is in good agreement with other study reported^14^.

**Figure 3:**
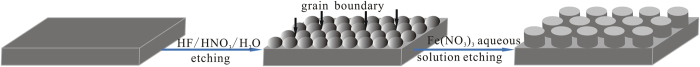
Schematic view of preparing porous Ag surface on the silver plated aluminum alloy sample by wet chemical etching.

Figure 3 shows the schematic view of preparing porous Ag by wet chemical etching. As known, Ag could be oxidized to silver nitrate in nitric acid solution, but the etching rate is slower. The etching process and reactive rate are promoted by adding HF into the nitric acid aqueous solution, the most probable reactions can be described by the following equations^16^


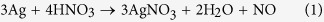



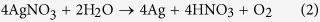



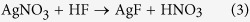



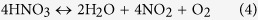


Second, Fe(NO_3_)_3_ exhibits a strong oxidizing property in ferric nitrate aqueous solution, which infiltrates into the grain boundary formed by acid mixture to enlarge the pores, obtaining the porous Ag surface.

**Figure 4:**
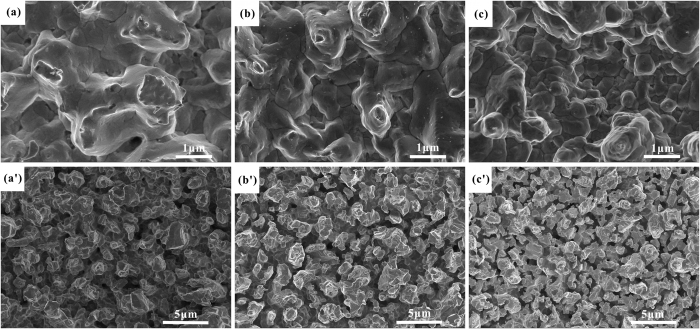
SEM images of Ag samples etched by acid mixture ((**a**) 5% HF, (**b**) 6% HF, (**c**) 7% HF) and the porous Ag surfaces etched by acid mixture and ferric nitrate aqueous solution sequentially ((**a′**) Ag-1:5% HF and 20% Fe(NO_3_)_3_, (**b′**) Ag-2:6% HF and 20% Fe(NO_3_)_3_, (**c′**) Ag-3:7% HF and 20% Fe(NO_3_)_3_).

**Figure 5:**
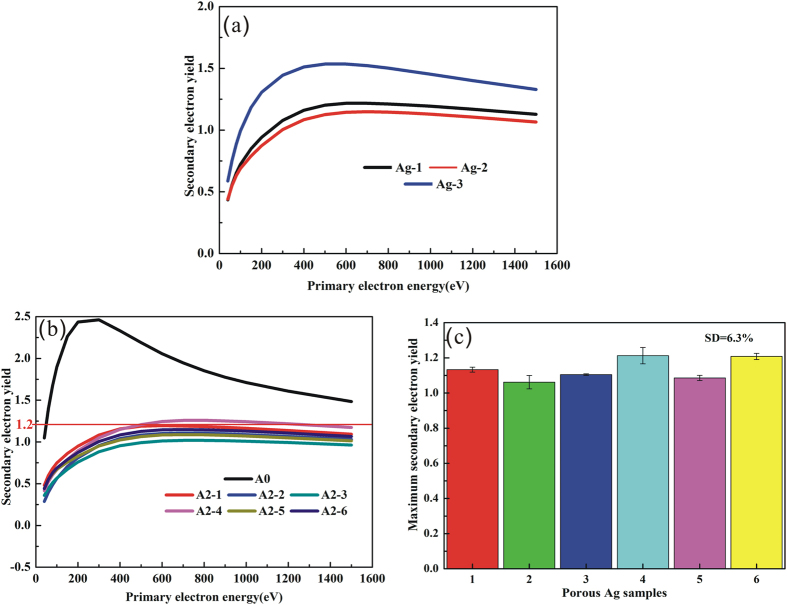
(**a**) SEY of the porous Ag surfaces. (**b**) SEY of original plated Ag sample and six research samples etched with the same process by 6% HF and 20% Fe(NO_3_)_3_ (A2-1-6), (**c**) Maximum SEY of the six research samples with the same process by 6% HF and 20% Fe(NO_3_)_3_ (A2-1-6).

According to the Monte-Carlo simulation of secondary electron trajectory, the larger ratio of depth to width and the greater porosity are, the lower SEY is. The porous surfaces of the samples etched by different acid mixture (5% HF, 6% HF and 7% HF) and ferric nitrate aqueous solution sequentially have been obtained. The results show that the porous Ag surfaces with high ratio of depth to length are all obtained by the three experiments (see Fig. 4a′–c). Upon volume percent of HF increasing from 5% to 7%, the crystal size of the first-step etched Ag surfaces decreases and the number of grain boundary increases (see Fig. 4a–c). The growing number of grain boundary results in the formation of smaller pores on the surfaces of the two-step etched samples. Besides, the pore parameters of the three porous Ag surfaces, measured by image processing method via Image J, are different (see Table 1). Furthermore, SEY of the three porous Ag surfaces is investigated, and with the porosity and pore counts increasing, the SEY is getting lower (see Fig. 5a). Electron trajectory in a single pore involves two processes: (1) primary electron enters into pores and excites secondary electron, (2) multiple reflections of the secondary electron occur in the pore and the pore wall to attenuate the secondary electron energy, which caused the trap of secondary electron. Hence much more pore counts and porosity are beneficial to reduce the SEY, and it is in accord with the research that the SEY suppression efficiency is sensitive to porosity of the cylindrical micro-pores array surface^26^.

**Figure 6:**
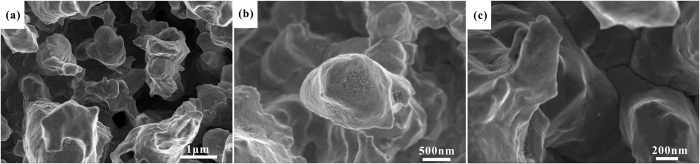
SEM images of TiO_2_ coatings obtained from EPLSD process on the porous Ag surface. (**a**) Magnification of 20 k. (**b**) Magnification of 30 k. (**c**) Magnification of 60 k.

The SEY coefficient of original plated Ag sample and six research samples etched with the same process by 6% HF and 20% Fe(NO_3_)_3_ sequentially can be seen from Fig. 6(b and c). The strong SEY suppression effect caused by the porous Ag surface can be observed. The maximum SEY of the porous Ag surface (Ag-2-1) is about 1.2, which means that a 52% reduction relative to the original Ag surface of maximum SEY 2.5. Six samples prepared by the same approach show the identically excellent multipactor suppression with SEY about 1.2, and the relative standard deviation (RSD) is 6.3% for six samples. This demonstrates that the stability and repeatability of the process are good.

### Characterization of TiO_2_ coatings on the porous Ag surface

SEM images with different magnifications of TiO_2_ coatings on the porous Ag surface are shown in Fig. 6. On the low magnification level (Fig. 6a), the surface morphology of TiO_2_ coatings exhibit the same porous structure as the two-step etched Ag samples. On the high magnification level, it is easy to see that the TiO_2_ coatings are formed of small particles with a size of 10 nm on both the bulges and holes of the porous Ag surface (Fig. 6b and c). Therefore, SEM images confirm that the uniform TiO_2_ coatings are prepared on the porous Ag surface.

**Figure 7:**
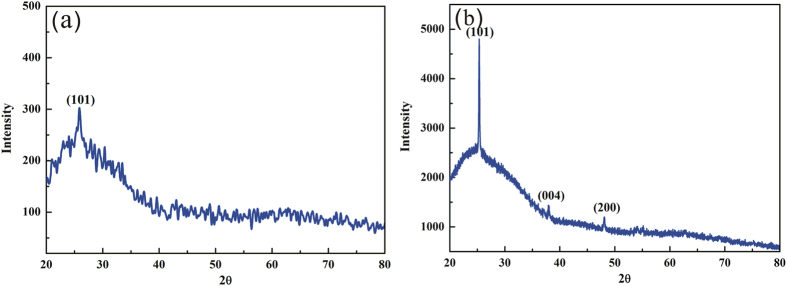
XRD pattern of TiO_2_ coatings obtained from the same EPLSD process on glass surface. (**a**) Under 5 wt% TBT, (**b**) under 20 wt% TBT.

To analyze the chemical composition and crystal type of the TiO_2_ coatings, the porous Ag/TiO_2_ surface was characterized by XRD. However, it is difficult to observe the diffraction peaks of TiO_2_ because of the strong diffraction peak of silver. Hence, TiO_2_ coatings were obtained from the same EPLSD process on the amorphous glass surface under 5 wt% TBT. The XRD pattern of TiO_2_ coatings in Fig. 7(a) shows only weak anatase peak (101) at 25.8°, we suspect that the TiO_2_ coatings are too thin, resulting in other anatase peaks invisible. To clarify this, TiO_2_ coatings were prepared by the same EPLSD process on the amorphous glass surface under 20 wt% TBT, which means four times more than that in the original procedure. The XRD pattern of TiO_2_ coatings in Fig. 7(b) is drawn to observe the composition and crystalline phase of the TiO_2_ coatings on the amorphous glass surface, the sample shows anatase peak (101) at 25.36°, peak (004) at 37.96° and peak (200) at 48.09°. This is similar to other studies reported^27^,^28^.

**Figure 8:**
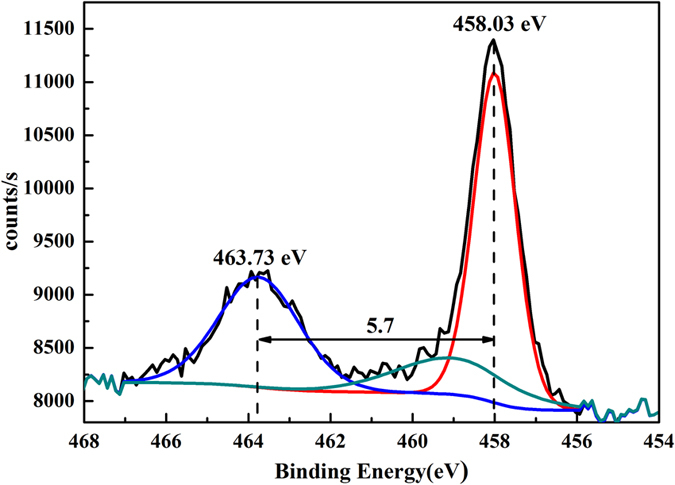
Ti_2p_ XPS spectra of TiO_2_ coatings obtained from EPLSD process on the porous Ag surface.

To further prove the composition and crystalline phase of the TiO_2_ coatings, the high-resolution XPS spectrum of Ti(2p) of the porous Ag/TiO_2_ surface was analyzed. Figure 8 shows that there are two main peaks in the Ti(2p) binding energy region, the peak located at binding energy of 463.73 eV is assigned to the Ti(2p_1/2_) and another one located at 458.03 eV corresponds to the Ti(2p_3/2_). The slitting between Ti(2p_1/2_) and Ti(2p_3/2_) core levels is 5.7 eV, indicating a normal state of Ti^4+^ in the anatase phase of TiO_2_ coatings^29^. It is interesting that the core levels of Ti(2p_1/2_) and Ti(2p_3/2_) show a 1.4–1.6 eV shift to lower binding energy compared with the corresponding bulk TiO_2_ materials(Ti(2p_1/2_) at 465.3 eV and Ti(2p_3/2_) at 459.4 eV). Ti^3+^ state appears at 459.1 eV by means of XPS-peak-differentation-imitating analysis, therefore, the lower binding energy is the result of defect chemical state of metal atoms from Ti^4+^ to Ti^3+^ in TiO_2_ coatings^30^,^31^.

**Figure 9:**
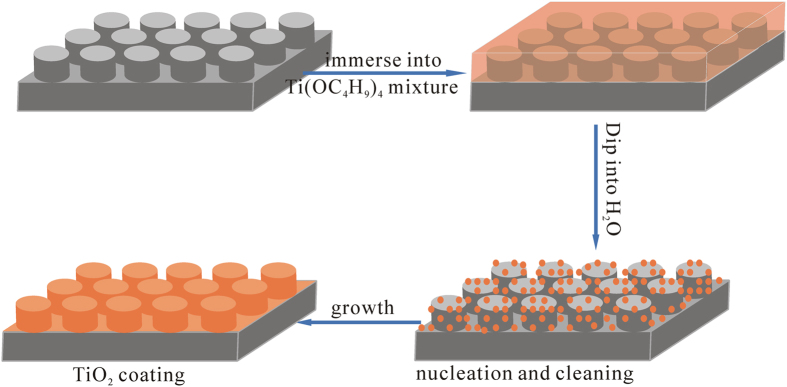
Schematic illustration of the EPLSD process for TiO_2_ coatings.

According to the above results, it is demonstrated that conformal and dense TiO_2_ films on the porous Ag surface are facilely synthesized by EPLSD. The possible mechanism of forming TiO_2_ coatings on the porous Ag surface by EPLSD process is described in Fig. 9. Firstly, the porous Ag surface adsorbs much more Ti(OC_4_H_9_)_4_ during immersing into Ti(OC_4_H_9_)_4_ mixture, the hydrolysis process is very slow due to complexant of acetic acid. After 20 min, there is excessive Ti(OC_4_H_9_)_4_ mixture covering the porous Ag surface. Secondly, the Ag surface with excessive Ti(OC_4_H_9_)_4_ mixture is dipped into deionized water for 10 min, which would react with H_2_O swiftly to produce a large number of TiO_2_ nucleus. Meanwhile, TiO_2_ nucleus grows uniformly into particles, which eventually form TiO_2_ coatings on the porous Ag surface. Deionized water plays an important role in the EPLSD process, which is responsible for both hydrolyzing Ti(OC_4_H_9_)_4_ to TiO_2_ and cleaning extra TiO_2_ particles of poor adhesion.

### Multipactor suppression of Au coatings prepared on the porous Ag/TiO_2_ surface

**Figure 10:**
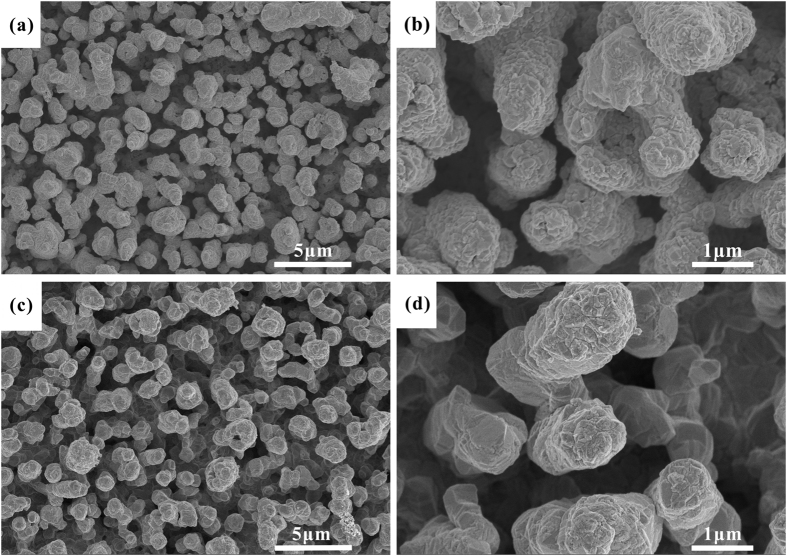
SEM images of Ag/TiO_2_/Au coatings (**a**) and Ag/Au coatings (**c**,**b** and **d**) are high magnification of (**a** and **c**).

Figure 10(a and b) display the Au coatings on the porous Ag/TiO_2_ surface. Au coatings show uniform aggregated particles, the size of the particles is about 200 nm. Fortunately, the porous Ag/TiO_2_ surface is covered completely by Au particles, and the micro-pores with higher aspect ratio and micrometer scale exist. However, when preparing directly Au coatings on porous Ag surface, continuous and integral Au films are formed (see Fig. 10c and d). Compared with porous Ag surface, the porosity of Ag/TiO_2_/Au coatings is decreasing significantly, but a slight reduction for the porosity of Ag/Au has been observed (see Table 2).

Accordingly, formation mechanisms of Au coatings on porous Ag/TiO_2_ and Ag surface are distinct. When deposition of Au coatings on porous Ag/TiO_2_ surface, the reduction of Au(III) is drastically accelerated at TiO_2_ surface since the resulting TiO_2_ particles on the etched Ag surface are active sites for the oxidation of glucose and the concurrent reduction of Au(III) to Au(0), while Au coatings are mainly formed by galvanic displacement on porous Ag surface. The surface morphology of Au coatings on porous Ag/TiO_2_ surface is porous and consistent with that of Ag/TiO_2_ coatings. It confirms that the reduction of Au^3+^ mostly occurs at TiO_2_ surface with no new particle nucleation. Agitation of the plating solution is essential for obtaining relatively homogeneous and close packed gold particles^32^.

**Figure 11:**
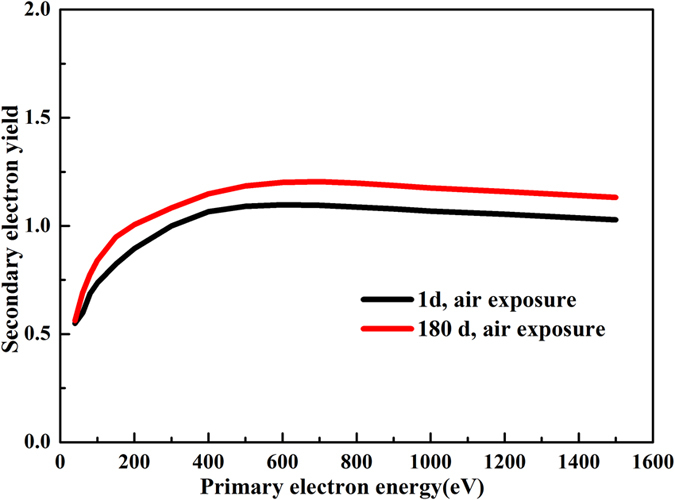
SEY of porous Ag/TiO_2_/Au coatings for 1 d and 180 d air exposure.

Figure 11 shows the SEY coefficient of porous Ag/TiO_2_/Au coatings for 1 d and 180 d air exposures. The SEY of the porous Ag/TiO_2_/Au coatings for 1 d air exposure is 1.1, and that of the porous Ag/TiO_2_/Au coatings for 180 d air exposure is only increased to 1.23. We had known that the SEY coefficient of 2 um Au on smooth Ag plating with short air exposure is above 1.8^10^. It is clear that the porous Ag/TiO_2_/Au coatings show pronounced SEY suppression effect when compared with smooth Ag/Au coatings. The range that an electron of a given primary energy below 2000 eV can penetrate through Au material is lower than 50 nm^16^,^33^, and thus much smaller than the Au layer thickness on either porous Ag/TiO_2_ surface or smooth Ag surface. Therefore, the structure of coatings plays a significant role in SEY suppression. We also presented the comparison of the maximum SEY of the porous surface between the study and the reported literature in Table 3, the maximum SEY of the porous Ag surface is lower than that reported in literature, moreover, the SEY suppression and environmental stability of porous Ag/TiO_2_/Au surface is more excellent.

## Discussion

From aforementioned results, we have demonstrated that porous Ag/TiO_2_/Au coatings can be prepared by two-step wet chemical etching, EPLSD and electroless plating, step by step. The SEY of Ag coatings depends on the porosity of the porous Ag surface, which are got by etching with different acid mixture and ferric nitrate aqueous solution sequentially. SEY measurements reveal that the porous Ag coatings etched by 6% HF and 20% Fe(NO_3_)_3_ sequentially exhibit stronger multipactor suppression with SEY below 1.2 and the two-step wet chemical etching processes are highly reproducible with the relative standard deviation (RSD) of 6.3%.

Anatase TiO_2_ thin films are prepared by a simple and effective EPLSD method, in which deionized water is crucial. It is responsible for both hydrolyzing Ti(OC_4_H_9_)_4_ to TiO_2_ and cleaning extra TiO_2_ particles with poor adhesion. TiO_2_ films act as not only transition layer but also active sites for the reduction of Au(III) to Au(0) via electroless plating.

Because the oxidation of porous Ag coatings are prevented with TiO_2_ and Au coatings, and the chemical and physical properties of Au coatings are stable to long term air exposure, which has an inherently lower SEY coefficient. Therefore, porous Ag/TiO_2_/Au coatings which are exposured in air for 180 d exhibit excellent mutipactor suppression with SEY 1.23.

## Methods

### Fabrication of porous Ag surface

A porous Ag surface was chemically etched on the silver plated aluminum alloy sample by an acid mixture and ferric nitrate aqueous solution sequentially. Prior to the wet chemical etching, surface contamination was removed by ultrasonication in detergent, deionised water, acetone and methanol for 15 min, respectively, and then dried out at 60 °C. Secondly, a different amount of HF (40 wt%, Tianjin Tianli Chemical Reagent Co. Tianjin, China, analytical reagents) was added into 25 mL deionised water, then 12.5 mL HNO_3_ (68 wt%, Xilong Chemical Co. Guangzhou, China, analytical reagents) was put in, and then the deionised water was added to complete a total volume of 50 mL. The volume percent of HF was varied from 5% to 7%. Then the silver plated aluminum alloy samples were totally immersed into the acid mixture for 3 min at 24 °C. The samples were then rinsed in abundant deionised water, using ultrasound, and desiccated at 60 °C. Thirdly, the samples were etched by using 20 wt% ferric nitrate (Guangzhou Huada Chemical Reagent Co. Guangzhou, China, analytical reagents) aqueous solution for 40 seconds at 50 °C, then the process was stopped by rinsing in abundant deionised water via ultrasound. Finally the samples were dried at 60 °C and wrapped in weighing paper for preservation.

### Fabrication of TiO_2_ coatings on porous Ag surface by electroless-plating-like solution deposition

TiO_2_ coatings on porous Ag surface were obtained by the reaction of tetrabutyl titanate (Ti(OC_4_H_9_)_4_, Tianjin Kermel Chemical Reagent Co. Tianjin, China, analytical reagents) mixture with deionised water. The Ti(OC_4_H_9_)_4_ mixture was prepared by adding tetrabutyl titanate to an ethanol solution of glacial acetic acid at the mass ratio of 0.5:0.4:10 (Ti(OC_4_H_9_)_4_:CH_3_COOH:C_2_H_5_OH). The porous Ag surface was first immersed into the Ti(OC_4_H_9_)_4_ mixture with stirring for 20 min, and then dipped quickly into 50 mL deionised water with stirring for 10 min, then the sample was dried for 30 min at 60 °C. Finally, the sample was calcined for 1 h at 400 °C.

### Fabrication of Au coatings on porous Ag/TiO_2_ surface by electroless plating

Au coatings were synthesized by electroless plating on the sample coated with porous Ag/TiO_2_. The electroless plating was performed for 1 h at 30 °C in the coating solution containing 4 mM chlorauric acid (HAuCl_4_), 8 mM glucose (C_6_H_12_O_6_) and sodium carbonate, sodium carbonate was employed to adjust the pH of the solution to 11.1. Then the sample was cleaned by ultrasonication in deionised water and dried for 30 min at 60 °C.

### Characterization

Field-emission scanning electron microscopy (SEM) was performed using Hitachi S-4800, which was used to investigate the surface morphology of the samples. The surface composition and elemental chemical state of the samples were examined by X-ray photoelectron spectroscopy (XPS) using a Model Axis Ultra (Kratos Analytical Ltd.) apparatus. The crystalline structure of the TiO_2_ was investigated by X-ray diffraction (XRD, D/max2200PC X-ray powder diffractometer, Japan) using CuKα radiation.

### SEY measurements

For the SEY measurements, the vacuum pressure was 10^−8^ Torr, a thermionic electron gun (Model DESA 150, Staib Instruments, German) supplied the incident or primary electron beam, the electron gun emitted electron beam with energy from 20 eV to 5000 eV. The secondary electron emission coefficient was tested by the conventional sample-current method. Firstly, the incident current *I*_p_ was measured when the sample was biased at +500 V, at which the incident electrons were completely absorbed, without secondary electrons escaping from the surface. Secondly, −20 V negative bias was applied to the sample, when the secondary electrons excited by the incident electron were considered to escaping from the sample surface, the current *I*_S_ was got. Therefore, the SEY was:


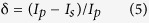


## Additional Information

**How to cite this article:** Wu, D. *et al*. Fabrication of Porous Ag/TiO_2_/Au Coatings with Excellent Multipactor Suppression. *Sci. Rep.* **7**, 43749; doi: 10.1038/srep43749 (2017).

**Publisher's note:** Springer Nature remains neutral with regard to jurisdictional claims in published maps and institutional affiliations.

## Figures and Tables

**Figure 1:**
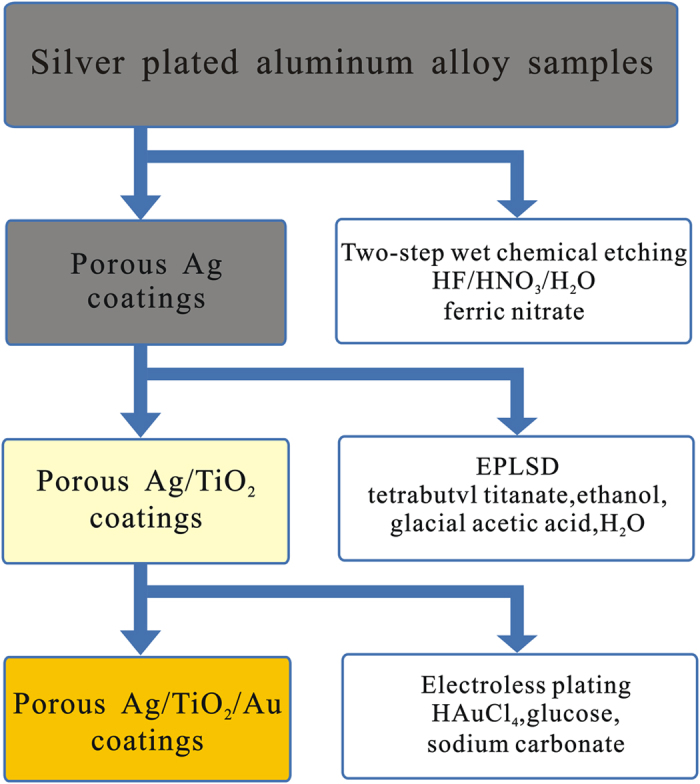
Scheme of the overall synthetic process developed to prepare Ag/TiO_2_/Au coatings.

**Table 1:** The comparison of pore parameters of the porous Ag surfaces.

Sample	Porosity (%)	Counts	Mean pore diameter (μm)
Ag-1	62.42	4434	0.114
Ag-2	63.90	5670	0.105
Ag-3	40.63	3729	0.156

**Table 2:** The comparison of different porosity of the porous Ag, Ag/Au and Ag/TiO_2_/Au samples.

Sample	Porosity (%)
Porous Ag	63.90
Porous Ag/Au	51.37
Porous Ag/TiO_2_/Au	38.07

**Table 3:** Comparison of the maximum SEY of the porous surface between the study and the reported literature.

Sample	Surface	Maximum SEY	Maximum SEY (air exposure)
S-1	Porous Ag	1.17	—
S-2	Porous Ag/TiO_2_/Au	1.10	1.23
R-1^16^	micro-structured Ag/Au	1.21	1.4
R-2^14^	micro-porous Ag	1.21	—
R-3^15^	Ag trap structures	1.53	—

S: samples in the study, R: samples in the reported literature, —: no investigation of environmental stability.

## References

[b1] Valizadeh R. . Low secondary electron yield engineered surface for electron cloud mitigation. Applied Physics Letters 105, 231605–231609 (2014).

[b2] Gvozdev A. K., Zharova N. A., Zaitsev N. I., Semenov V. E. & Sorokin A. A. Development of a Multipactor Discharge in the Output Channel of a Powerful Pulsed Gyroklystron. Technical Physics 57, 1394–1399 (2012).

[b3] Hueso J. . Study of the Multipactor Effect in Bandpass Wedge-Shaped Waveguide Filters. IEEE Transactions on Electron Devices 58, 3205–3212 (2011).

[b4] Ripalda J. M., Monteroa I., Vázquez L., Raboso D. & Galán L. Secondary electron emission and photoemission studies on surface films of carbon nitride. Journal of Applied Physics 99, 043513–043519 (2006).

[b5] Larciprete R., Grosso D. R., Trolio A. D. & Cimino R. Evolution of the secondary electron emission during the graphitization of thin C films. Applied Surface Science 328, 356–360 (2015).

[b6] Ruiz A. . UHV reactive evaporation growth of titanium nitride thin films, looking for multipactor effect suppression in space applications. Vacuum 81, 1493–1497 (2007).

[b7] Malgras V. . Nanoarchitectures for Mesoporous Metals. Advanced Materials 28, 993–1010 (2016). 26515804 10.1002/adma.201502593

[b8] Lin J. J. . Mesoporous anatase single crystals for efficient Co^(2+/3+)^-based dye-sensitized solar cells. Nano Energy 11, 557–567 (2015).

[b9] Sun L., Campbell M. G. & Dinca M. Electrically Conductive Porous Metal -Organic Frameworks. Angew. Chem. Int. Ed 55, 3566–3579 (2016). 10.1002/anie.201506219 26749063

[b10] Sakaushi K. & Antonietti M. Carbon- and Nitrogen-Based Porous Solids: A Recently Emerging Class of Materials. Bull. Chem. Soc. Jpn 88, 386–398 (2015).

[b11] Zhu C. Z., Li H., Fu S. F., Du D. & Lin Y. H. Highly efficient nonprecious metal catalysts towards oxygen reduction reaction based on three-dimensional porous carbon nanostructures. Chem. Soc.Re. 45, 517–531 (2016). 10.1039/c5cs00670h 26658546

[b12] Malgras V. . Templated Synthesis for Nanoarchitectured Porous Materials. Bull. Chem. Soc. Jpn 88, 1171–1200 (2015).

[b13] Yang S. C. & Luo X. Mesoporous nano/micro noble metal particles: synthesis and applications. Nanoscale 6, 4438–4457 (2014). 24676151 10.1039/c3nr06858g

[b14] Ye M. . Investigation into anomalous total secondary electron yield for porous Ag surface under oblique incidence conditions. Journal of Applied Physics 114, 104905–104912 (2013).

[b15] Bao Y., Zhang Y. H., Ma J. Z., Zhao Y. R. & Wu D. D. Controllable fabrication of one-dimensional ZnO nanoarrays and their application in constructing silver trap structures. RSC Advances 63, 33198–33205 (2014).

[b16] Nistor V. . Multipactor suppression by micro-structured gold/silver coatings for space applications. Applied Surface Science 315, 445–453 (2014).

[b17] Chen C., Cheng Y., Dai Q. L. & Song H. W. Radio Frequency Magnetron Sputtering Deposition of TiO_2_ Thin Films and Their Perovskite Solar Cell Applications. Scientific Reports 5, 17684–17690 (2015). 26631493 10.1038/srep17684 PMC4668551

[b18] Jeong H. Y., Lee J. Y. & Choi S. Y. Interface-Engineered Amorphous TiO_2_-Based Resistive Memory Devices. Advanced Functional Materials 20, 3912–3917 (2010).

[b19] Buso D., Pacifico J., Martucci A. & Mulvaney P. Gold-Nanoparticle-Doped TiO_2_ Semiconductor Thin Films: Optical Characterization. Advanced Functional Materials 17, 347–354 (2007).

[b20] Wu L. Z., Yu Y. & Zhi J. F. Low cost and large-area fabrication of self-cleaning coating on polymeric surface based on electroless-plating-like solution deposition approach. RSC Advances 5, 10159–10164 (2015).

[b21] Wu L. Z., Yu Y., Song L. & Zhi J. F. M/TiO_2_ (M = Au, Ag) transparent aqueous sols and its application on polymeric surface antibacterial post-treatment. Journal of Colloid and Interface Science 446, 213–217 (2015). 25678155 10.1016/j.jcis.2015.01.050

[b22] Kim D. Characterization of TiO_2_/Au/TiO_2_ films deposited by magnetron sputtering on polycarbonate substrates. Applied Surface Science 257, 704–707 (2010).

[b23] Wang X. X., Yang T., Li X. & Jiao K. Three-step electrodeposition synthesis of self-doped polyaniline nanofiber-supported flower-like Au microspheres for high-performance biosensing of DNA hybridization recognition. Biosensors and Bioelectronics 26, 2953–2959 (2011). 21185714 10.1016/j.bios.2010.11.045

[b24] Menzel H., Mowery M. D., Cai M. & Evans C. E. Surface-Confined Nanoparticles as Substrates for Photopolymerizable Self-Assembled Monolayers. Advanced Materials 11, 131–134 (1999).

[b25] Whelan J. C. . Electroless Plating of Thin Gold Films Directly onto Silicon Nitride Thin Films and into Micropores. ACS Appl. Mater. Interfaces 6, 10952–10957 (2014). 24999923 10.1021/am501971n

[b26] Ye M. . Suppression of secondary electron yield by porous array structure. Journal of Applied Physics 113, 074904–074911 (2013).

[b27] Moon J., Hedman H. P., Kemell M., Tuominen A. & Punkkinen R. Hydrogen sensor of Pd-decorated tubular TiO_2_ layer prepared by anodization with patterned electrodes on SiO_2_/Si substrate. Sensors and Actuators B: Chemical 222, 190–197 (2016).

[b28] Jongyun M. . A study of monitoring hydrogen using mesoporous TiO_2_ synthesized by anodization. Sensors and Actuators B: Chemical 189, 246–250 (2013).

[b29] Metin Y. . Fabrication and characterization of nanostructured anatase TiO_2_ films prepared by electrochemical anodization and their photocatalytic properties. Journal of Alloys and Compounds 651, 59–71 (2015).

[b30] Sun Z. Q. . Generalized self-assembly of scalable two-dimensional transition metal oxide nanosheets. Nature Communications 5, 3813–3813 (2014). 10.1038/ncomms4813 24814859

[b31] Wendt S. . The role of interstitial sites in the Ti*3d* defect state in the band gap of titania. Science 320, 1755–1759 (2008). 18535207 10.1126/science.1159846

[b32] Hrapovic S., Liu Y., Enright G., Bensebaa F. & Luong H. T. New Strategy for Preparing Thin Gold Films on Modified Glass Surfaces by Electroless Deposition Langmuir. 19, 3958–3965 (2003).

[b33] Wilson G. & Dennison J. R. Approximation of Range in Materials as a Function of Incident Electron Energy. IEEE Transactions on Plasma Science 40, 291–297 (2012).

